# Bridging the gap: Integrating cutting-edge techniques into biological imaging with deepImageJ

**DOI:** 10.1017/S2633903X24000114

**Published:** 2024-11-22

**Authors:** Caterina Fuster-Barceló, Carlos García-López-de-Haro, Estibaliz Gómez-de-Mariscal, Wei Ouyang, Jean-Christophe Olivo-Marin, Daniel Sage, Arrate Muñoz-Barrutia

**Affiliations:** 1 Bioengineering Department[CMT1], Universidad Carlos III de Madrid, Leganes, Spain; 2 Bioengineering Division, Instituto de Investigación Sanitaria Gregorio Marañón, Madrid, Spain; 3 Biological Image Analysis Unit, Institut Pasteur, Paris, France; 4 Optical Cell Biology Group, Instituto Gulbenkian de Ciência, Oeiras, Portugal; 5 Science for Life Laboratory, Department of Applied Physics, KTH Royal Institute of Technology, Stockholm, Sweden; 6 Biological Image Analysis Unit, Institut Pasteur, Centre National de la Reserche Scientifique UMR3691, Université Paris Cité, París, France; 7 Biomedical Imaging Group and Center for Imaging, Ecole Polytechnique Fédérale de Lausanne (EPFL), Lausanne, Switzerland

**Keywords:** BioImage model zoo, biological imaging, ImageJ, JDLL

## Abstract

This manuscript showcases the latest advancements in deepImageJ, a pivotal Fiji/ImageJ plugin for bioimage analysis in life sciences. The plugin, known for its user-friendly interface, facilitates the application of diverse pre-trained convolutional neural networks to custom data. The manuscript demonstrates several deepImageJ capabilities, particularly in deploying complex pipelines, three-dimensional (3D) image analysis, and processing large images. A key development is the integration of the Java Deep Learning Library, expanding deepImageJ’s compatibility with various deep learning (DL) frameworks, including TensorFlow, PyTorch, and ONNX. This allows for running multiple engines within a single Fiji/ImageJ instance, streamlining complex bioimage analysis workflows. The manuscript details three case studies to demonstrate these capabilities. The first case study explores integrated image-to-image translation followed by nuclei segmentation. The second case study focuses on 3D nuclei segmentation. The third case study showcases large image volume segmentation and compatibility with the BioImage Model Zoo. These use cases underscore deepImageJ’s versatility and power to make advanced DLmore accessible and efficient for bioimage analysis. The new developments within deepImageJ seek to provide a more flexible and enriched user-friendly framework to enable next-generation image processing in life science.

## Impact Statement

The advancements in deepImageJ, detailed in this paper, represent a significant leap in bioimage analysis, crucial for life sciences. By enhancing this Fiji/ImageJ plugin, the research bridges the gap between complex deep learning (DL) models and practical applications, making advanced bioimage analysis accessible to a broader audience. The integration of the Java Deep Learning Library (JDLL) within deepImageJ is particularly remarkable, as it expands compatibility with updated DL frameworks. This allows for the seamless execution of multiple models in a single instance of Fiji/ImageJ, simplifying the construction and automation of complex image analysis pipelines. The implications of this research are far-reaching, extending beyond academic circles to potentially impact various sectors, including healthcare, pharmaceuticals, and biotechnology. The enhanced capabilities of deepImageJ in handling intricate DL pipelines, with large volumetric image data, facilitate a sophisticated and efficient analysis of biological data. Such advancements are vital for accelerating research and development in biomedical imaging, drug discovery, and understanding complex biological processes. This contribution aims to offer a tool that empowers researchers, irrespective of their computational expertise, to leverage advanced technologies in the analysis of their biomedical images. The wide applicability and ease of use of deepImageJ have the potential to foster interdisciplinary collaborations, drive innovation, and facilitate discoveries across various scientific and industrial sectors.

## 1. Introduction

Bioimage analysis has undergone remarkable progress due to the advent of open-source tools, making advanced technologies more accessible. These tools include BiaPy,^(^^1^^)^ CellPose,^(^^2^^)^ CellProfiler,^(^^3^^)^ Icy,^(^^4^^)^ Ilastik,^(^^5^^)^ ImJoy,^(^^6^^)^ Napari,^(^^7^^)^ QuPath,^(^^8^^)^ ZeroCostDL4Mic,^(^^9^^)^ and others. One notable development in this field is the emergence of zero-code tools, which streamline the integration of complex analysis pipelines and DL networks across various bioimage analysis domains. A key example is Fiji/ImageJ,^(^^10^^)^ an open-source desktop application central to bioimage analysis. It offers extensive capabilities enhanced by a vibrant community that develops plugins, enabling tasks ranging from basic image processing to the application of advanced DL networks specialized in star-convex object segmentation (i.e., nuclei).^(^^11^^)^

DeepImageJ,^(^^12^^)^ a freely available plugin for Fiji, stands out in the realm of zero-code toolkits. As of May 2024, it has been downloaded more than 60,000 times. It provides an integrated environment within Fiji for executing third-party models from DL libraries. Notably, deepImageJ is a recognized community partner of the BioImage Model Zoo (https://bioimage.io),^(^^13^^)^ hosting pre-trained models for life sciences. The streamlined installation process of deepImageJ, coupled with its intuitive user interface, is designed to lower the entry barrier and simplify the execution of complex DL pipelines, making advanced bioimage analysis more accessible to biologists. DeepImageJ’s practicality is demonstrated in effective workflows for microscopy image analysis.^(^^14^^)^

This manuscript presents deepImageJ 3.0, the latest version of deepImageJ leveraging the BioImage Model Zoo’s strengths and introducing the JDLL.^(^^15^^)^ This new version of deepImageJ brings notable improvements, including enhanced integration with the BioImage Model Zoo, increased compatibility with various DL frameworks, and the ability to handle larger images. These advancements make deepImageJ a more versatile and powerful tool in the Fiji/ImageJ ecosystem, especially for the application of complex image analysis tasks, marking a significant advancement in accessible tools for bioimage analysis. The case studies included in this manuscript exemplify the practical applications of these improvements, showcasing deepImageJ 3.0’s enhanced capabilities in diverse bioimage analysis scenarios.

## 2. Advancements in deepImageJ 3.0: Expanding capabilities in bioimage analysis

With the recent update of deepImageJ (deepImageJ 3.0), a range of significant advancements have arisen, thereby expanding deepImageJ’s functionalities and broadening its applicability in bioimage analysis. These new features are designed to simplify the integration and execution of DL models, offering researchers a more versatile and efficient toolset.

### 2.1. JDLL: A comprehensive toolkit

An important feature of deepImageJ 3.0 is its integration with the JDLL.^(^^15^^)^ JDLL acts as an all-encompassing toolkit and application programming interface, facilitating the creation of sophisticated scientific application and image analysis pipelines with DL functionality. This library simplifies the complex tasks of installing, maintaining, and executing DL models, with support for major frameworks like TensorFlow, PyTorch, and ONNX. The DL engine installer and DL model runner within JDLL provide an intuitive workflow for downloading, integrating, and performing inference, offering a harmonized approach to utilizing various DL frameworks within the Fiji/ImageJ ecosystem. This synergy between deepImageJ and JDLL significantly enhances the ability to execute DL models within Fiji/ImageJ without intricate installations, offering researchers a more streamlined and cohesive environment for bioimage analysis.^(^^15^^)^

Additionally, JDLL enhances deepImageJ by integrating the latest DL frameworks, updating and expanding those that are compatible with previous versions. The field of DL is rapidly advancing; therefore, staying updated with the corresponding frameworks is crucial. The integration of JDDL as a back-end provides deepImageJ with the capacity to deliver the most recent state-of-the-art methodologies to its end users.

### 2.2. Multiple engine compatibility: Running different engines in a single Fiji/ImageJ instance

A substantial advancement in deepImageJ 3.0 is its newfound capability to load and unload multiple DL frameworks within the same Fiji/ImageJ instance. This development allows for the building of image analysis pipelines that incorporate multiple DL stages, utilizing different engines. This improved compatibility enables seamless integration of models developed in TensorFlow, PyTorch, and ONNX, creating a unified workflow. Such an enhancement provides users with the flexibility to execute a variety of models in a single, integrated pipeline. An example of this is presented in Case Study 1, where image-to-image translation and cell segmentation are performed concurrently within the same Fiji/ImageJ instance.

### 2.3. Extended framework compatibility: Supporting various versions of DL frameworks

DeepImageJ 3.0 is compatible with the BioImage Model Zoo, which broadens the range and diversity of accessible executable pre-trained models.

A significant aspect of this advancement is the first-class support for the BioImage Model Zoo models, designed to facilitate the seamless sharing and reproduction of DL models for bioimage analysis. The BioImage Model Zoo serves as a centralized repository, accessible through the BioImage.IO website, where developers can upload trained DL models for bioimage analysis, along with comprehensive metadata and usage guidelines detailed in the model Resource Description File. This structured format ensures that each model is documented with all necessary information for its successful application across model consumer software.

Through the integration of the BioImage Model Zoo via deepImageJ, a standarized community-driven approach to model sharing and documentation is offered to the Fiji ecosystem. In addition, by adhering to the bioimage.io format, models gain a level of interoperability and documentation that simplifies their adoption across various software environments, not limited to deepImageJ. This ecosystem-wide compatibility empowers both developers, by broadening the reach and impact of their work, and users, by providing access to a vetted collection of models ready for application in their scientific inquiries. Consequently, deepImageJ’s role extends beyond a mere execution platform to become a catalyst for community collaboration and innovation within the bioimage analysis domain.

In two of the three case studies used to demonstrate the performance of deepImageJ, we utilized BioImage Model Zoo models that require specific post-processing steps outside of the deepImageJ plugin. This highlights the ability of deepImageJ to run models end-to-end, which can be affected by the pre- and post-processing requirements of a specific model. By default, deepImageJ supports several types of post-processing, including binarization, scaling, mean calculation, and standardization. However, model-specific post-processings, such as those used by StarDist, may require user intervention or additional image processing steps, often accessible within the Fiji/ImageJ ecosystem. Typically, any special pre- or post-processing needed can be included within the Bioimage.IO model as an ImageJ macro file. Users will then need to create a workflow or macro that integrates both deepImageJ and this macro file.

### 2.4. Handling of big images enhancement: Leveraging ImgLib2

DeepImageJ 3.0, built upon ImgLib2,^(^^16^^)^ is equipped to handle large images, thereby demonstrating its capacity for a more efficient processing of extensive data sets within some limits, depending on the computer’s capability. The integration of ImgLib2 significantly boosts the flexibility and scalability of deepImageJ, making it adept at accommodating the large image sizes often encountered in bioimage analysis. This feature together with its tiling strategy,^(^^12^^)^ ensures that researchers can apply DL models to a wide array of image data, enabling thorough and detailed analyses.

In prior iterations, deepImageJ utilized a tiling strategy that facilitated the processing of large images on CPUs with limited resources. This was achieved by segmenting the original image into tiles, processing each tile individually, and subsequently reassembling them to produce the processed output of the original image. Thanks to the incorporation of ImgLib2,^(^^16^^)^ the tiling approach is enhanced to optimize memory management significantly and enable more efficient processing of large images compared to earlier versions. Generally, images up to one-tenth of the computer’s RAM are now manageable with deepImageJ.

Nonetheless, it is important to acknowledge that the maximum manageable image size is ultimately constrained by the computer’s hardware capabilities, as well as the size and complexity of the DL model employed.

## 3. Case studies

In this section, we showcase real-world applications of deepImageJ 3.0 through a series of case studies. These studies highlight the software-enhanced features, as previously discussed. Through these practical examples, our goal is to demonstrate the significant impact and versatility of deepImageJ in tackling various challenges encountered in bioimage analysis.

The first two cases would not have been feasible in earlier iterations of deepImageJ, as the versions of the DL frameworks required were too modern and lacked support at that time. With the integration of JDLL in deepImageJ 3.0, this is no longer an issue, as the software can now keep up with the latest DL frameworks and provide end-users with access to the most advanced and accurate methods available.

To construct these use cases, we employed two key software ecosystems. First is the BioImage Model Zoo, for which we have highlighted the integration with deepImageJ as essential for ensuring the reproducibility of analyses and the seamless acquisition of DL models within deepImageJ. The second ecosystem is ZeroCostDL4Mic, a suite of Google Colab notebooks designed to democratize DL model fine-tuning. ZeroCostDL4Mic minimizes the coding barrier, offering a user-friendly platform for researchers to adapt DL models to their specific needs without requiring extensive programming knowledge. These tools collectively demonstrate deepImageJ’s capability to function within a broader computational workflow, thereby expanding its utility and application in the biological imaging community.

### 3.1. Case study 1: deepImageJ pipeline for integrated image-to-image translation and nuclei segmentation

This case study showcases the advanced capabilities of deepImageJ, particularly its proficiency in integrating and executing diverse DL approaches, often challenged by library and dependency incompatibilities within a typical Python environment. Specifically, we have reproduced a sophisticated bioimage analysis pipeline that combines the creation of artificially labeled nuclei images from membrane staining images with subsequent nuclei segmentation.^(^^9^^)^ This approach allows us to generate synthetic nuclei images, which are easier to process, from an input image (stained cell membranes) that is more suitable for live imaging due to lower phototoxicity compared to direct nuclear staining.

DeepImageJ 3.0 utilizes its enhanced features to combine two distinct DL networks: Pix2Pix^(^^17^^)^ and StarDist.^(^^18^^)^ This integration enables the conversion of membrane-stained images to nuclei stains using Pix2Pix, exported in Pytorch 2.0.1, followed by nuclei segmentation with StarDist, implemented in TensorFlow 2.14. This case study not only demonstrates deepImageJ’s capacity to integrate diverse approaches but also highlights its ability to run models with different engines, addressing the often encountered incompatibility of libraries and dependencies in Python environments.^(^^9^^)^ The pipeline effectively manages and executes these models, each requiring distinct engines, showcasing deepImageJ’s versatility in handling complex bioimage analysis tasks.

**Figure 1.**
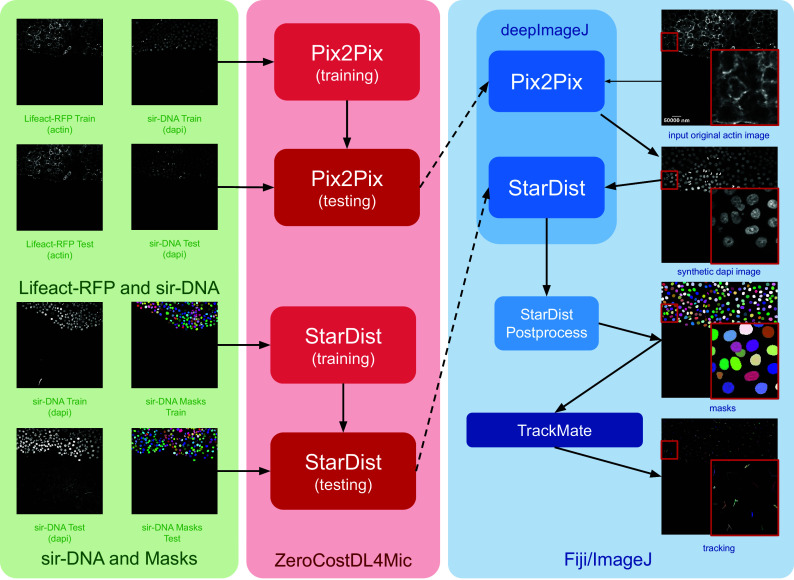
**Case Study 1: Image-to-image translation and cell segmentation: Pipeline and dataset.** The pipeline involves three main stages as follows: data set preparation, model training using ZeroCostDL4Mic, and inference and post-processing in deepImageJ. Initially, Pix2Pix and StarDist are fine-tuned with specific data sets. Pix2Pix transforms actin images into synthetic DAPI images, while StarDist creates masks from DAPI images. Once trained, the models are exported to the BioImage Model Zoo format and subsequently installed in deepImageJ. In the Fiji/ImageJ and deepImageJ environment, the pipeline first uses Pix2Pix to transform actin images into synthetic DAPI images, followed by the application of StarDist for nuclei segmentation. Finally, TrackMate is utilized for a thorough evaluation of cell tracking. A contrast enhancement has been applied to actin images for visualization purposes.

The construction of this pipeline is depicted in Figure 1. It comprises two main phases as follows: (i) fine-tuning both networks using ZeroCostDL4Mic (in red) and exporting them to the bioimage.io format; and (ii) performing inference with deepImageJ in Fiji (blue box). Within the deepImageJ environment, an ImageJ macro is used to process the five available time points of Lifeact-RFP images with Pix2Pix. This step results in synthetic SiR-DNA images, which effectively stain the nuclei. Subsequently, these images undergo processing with the deepImageJ implementation of StarDist, which involves applying the UNet model (trained via ZeroCostDL4Mic) in Fiji/ImageJ and the corresponding post-processing for nuclei segmentation to produce masks. These masks, obtained from five distinct time points, are then tracked using TrackMate,^(^^19^^)^ an ImageJ plugin, to visualize cell trajectories and track cell movement.

### 3.2. Case study 2: Comprehensive three-dimensional (3D) nuclei segmentation with deepImageJ

Case Study 2 emphasizes the capabilities of deepImageJ, particularly benefiting from its integration within the extensive image-processing ecosystem of Fiji/ImageJ. This integration affords the flexibility to run advanced pipelines automatically, including 3D+t image analysis, in a user-friendly manner. In particular, Case Study 2 demonstrates the segmentation of nuclei in microscopy images of whole embryos.

For enhanced reproducibility and to accommodate users without access to high-powered computational resources, this pipeline is executed in two-dimensional (2D), using a lightweight framework. By employing StarDist 2D (UNet model + postprocessing) and then applying MorphoLibJ^(^^20^^)^ connected components in 3D, we successfully mimic 3D segmentation. This approach demonstrates how the integration of deepImageJ into the Fiji/ImageJ ecosystem facilitates complex image analysis tasks, bypassing the need for extensive computational power typically required for direct 3D processing in bioimage analysis.

**Figure 2.**
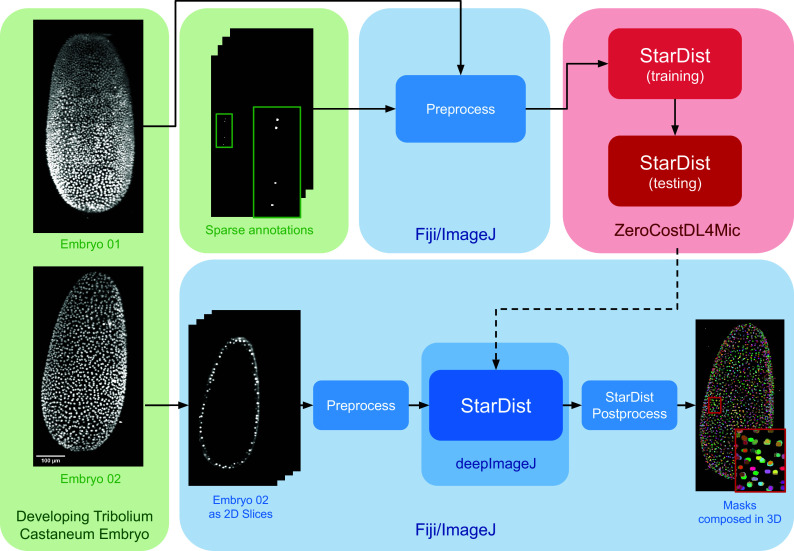
**Case Study 2: Three-dimensional (3D) nuclei segmentation: Pipeline and data set.** The data set consists of two distinct embryos, labeled 01 and 02. One embryo is used for fine-tuning the StarDist network in ZeroCostDL4Mic, following downsampling and noise filtering, whereas the other is utilized for inference. After training the StarDist model, it is employed in deepImageJ to create the masks, followed by StarDist postprocessing. The pipeline is completed with the application of Connected Components for 3D visualization. All 3D volumes are displayed as Z-projections.

The data set for this study is part of the Cell Tracking Challenge repository, specifically the “Developing *Tribolium castaneum* embryo”.^(^^21^^)^ This data set provides 3D volumetric data of two beetle embryos (Embryo 01 used in fine-tuning and Embryo 02 saved for testing), with accompanying sparse nuclei annotations of the beetle’s blastoderm at the junction of embryonic and extra-embryonic tissues. Several preprocessing steps are undertaken to leverage deepImageJ’s capabilities for running StarDist 2D. Initially, a targeted selection of slices from various time points in embryo 01 was conducted, guided by the availability of ground truth data within the data set from the Cell Tracking Challenge. This selection process was governed by the necessity to choose slices for which ground truth data existed, as these were imperative for the training phase. These images and masks are then downsampled to obtain a less demanding pipeline in terms of memory usage that can be reproduced across different computational systems. Following this, a median filter (kernel size, 7 pixels) is applied to reduce noise in the input images. All these preprocessing steps are done in the Fiji ecosystem. Then, moving to Google Colab, the prepared pair of images is processed using the StarDist notebook within the ZeroCostDL4Mic repository, as illustrated in Figure 2.

In Case Study 2, after the UNet model is fine-tuned, it is exported and integrated into deepImageJ as a bioimage.io model. The subsequent analysis in Fiji/ImageJ involves a structured approach as follows: (i) implementing preprocessing steps that mirror those used during the model’s training, (ii) deploying the StarDist model for inference, and (iii) applying a series of post-processing techniques for assessment and visualization. This includes downsampling and denoising of selected time points from Embryo 02, following the methodology utilized for the training dataset in Embryo 01). Each 2D slice of the embryo is then processed through the trained network. The final step involves enhancing the segmentation masks using the StarDist post-processing pipeline and applying MorpholibJ’s Connected Components^(^^20^^)^ for comprehensive 3D visualization of the nuclei.

### 3.3. Case study 3: Segmentation of *Arabidopsis* apical stem cells and integration with the BioImage model zoo in deepImageJ

In this use case, we highlight two key capabilities of deepImageJ: (i) its adeptness in handling large 3D images and (ii) its seamless integration with the BioImage Model Zoo.

The implementation of this pipeline involves using the 3D UNet *Arabidopsis* Apical Stem Cells model from the bioimage.io website,^(^^22^^)^ paired with the data set titled “*Research data supporting cell size and growth regulation in the Arabidopsis thaliana apical stem cell niche*”.^(^^23^^)^ This approach establishes an efficient yet robust pipeline for cell segmentation within apical stem cells, particularly focusing on the epidermal cell volumes in the apical meristem, using the 3D UNet pre-trained model from the BioImage Model Zoo.

**Figure 3.**
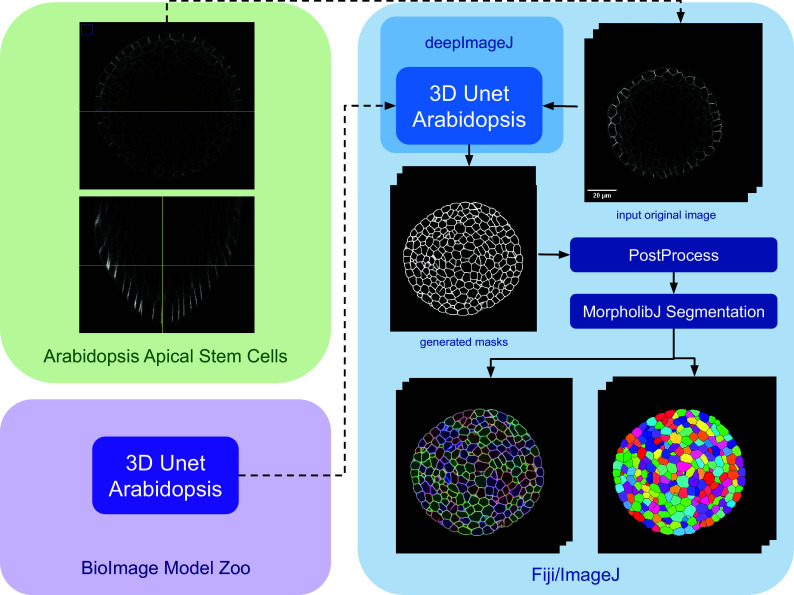
**Case Study 3: Segmentation of Arabidopsis apical stem cells: pipeline.** This diagram illustrates the pipeline for Case Study 3. Initially, the data set is acquired, followed by downloading and installing the model from the BioImage Model Zoo into deepImageJ. Subsequently, the model is applied to a selected root volume to generate a mask. The process concludes with post-processing and MorpholibJ segmentation to display catchment and overlay basins on the segmented image.

The main steps of the pipeline are summarized in Figure 3. Initially, the model is downloaded from the bioimage.io website and installed via the deepImageJ Install mode, changing the software or ecosystem where we are working from the BioImage Model Zoo to Fiji as indicated by the color change in Figure 3. Subsequently, a relatively large image, measuring 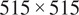 pixels with 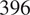 slices and representing a 3D volume of Plant 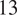 of the data set (chosen for its significant size), is selected for the analysis. The 3D UNet is then employed, and using the tiling strategy of deepImageJ,^(^^12^^)^ the image is processed in 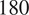 patches of 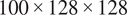 pixels to cover the whole 3D volume. After the model execution results in a segmentation mask, we apply a standardized post-processing procedure to refine the segmentation outcomes. Specifically, we enhance the contrast of the segmented membranes by applying a Gamma correction with a coefficient of 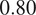 . Following this, we employ the morphological segmentation tool from MorphoLibJ,^(^^20^^)^ to further delineate the cell boundaries. This involves a sequence of morphological operations designed to prepare the image for precise segmentation, culminating in the application of a watershed algorithm set to a low tolerance threshold. This approach ensures the segmentation accuracy is maximized, leveraging the capabilities of MorphoLibJ to achieve refined segmentation results.

## 4. Discussion

The advancements presented in this manuscript reflect a significant leap in the capabilities of deepImageJ, a key plugin for Fiji/ImageJ in the domain of bioimage analysis. This tool evolution has the potential to positively impact the life sciences community, where the need for accessible, efficient, and versatile image analysis tools is ever-growing. Integrating deepImageJ with the BioImage Model Zoo and incorporating the JDLL underscore its role as a bridge between complex DL models and practical, user-friendly applications. Moreover, the seamless integration with the JDLL enhances the software’s capabilities, providing a unified platform for deploying diverse DL models.

**Figure 4.**
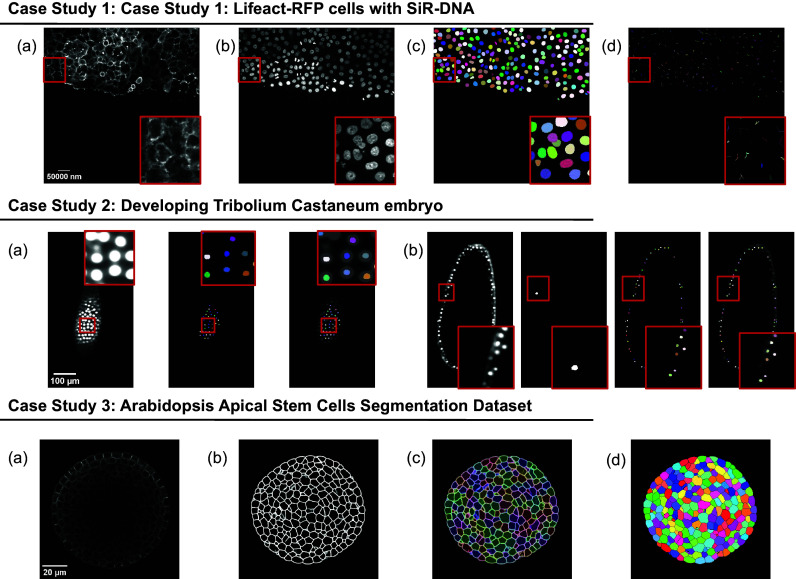
**Summary of the three case studies.** This figure provides an overview of three distinct case studies, highlighting deepImageJ’s versatility and integration with other tools and plugins. Case Study 1 illustrates the transformation of an actin membrane stain image (a) into a synthetic nuclei stain (b) image via Pix2Pix, followed by StarDist nuclei segmentation (c) and TrackMate cell tracking (d). Case Study 2 presents two examples of a single slice from input volume and StarDist output, with one including the Ground Truth (b). Case Study 3 shows the pipeline stages: (a) input image, (b) mask generation, (c) overlay of Morphological Segmentation basins, and (d) visualization of catchment basins.

The presented case studies demonstrate the profound adaptability and enhanced functionality of deepImageJ. A summary of these case studies is depicted in Figure 4. The first case study, focusing on image-to-image translation and nuclei segmentation, illustrates the software’s ability to integrate and execute multiple DL environments within a single Fiji/ImageJ instance. This capability is crucial in biological contexts where multifaceted analysis is often required. It facilitates the researchers to delve into intricate biological pipelines without the need for extensive coding expertise. The second case study further showcases the power of the integration of deepImageJ into the Fiji/ImageJ ecosystem to, in this case, handle complex 3D nuclei segmentation, a task that is increasingly relevant as imaging technologies advance. Finally, the third case study emphasizes the tool’s adeptness in processing large 3D images, an essential feature for analyzing extensive data sets commonly encountered in modern biological research as well as the deepImageJ integration with the BioImage Model Zoo.

With this new version of deepImageJ, significant improvements have been made in the execution of the workflow as demonstrated in the case studies. It introduces previously unavailable features, streamlining the processes and pipelines showcased in this paper. Namely, the new support for PyTorch 2 and TensorFlow 2 has made possible the execution of case studies 1 and 2, which was not possible before.

The case studies presented illustrate the versatility of deepImageJ and its ability to be adapted for various biological applications. For instance, in Use-case 1, we demonstrated image translation with Pix2Pix for actin-nucleus translation in cell culture, which could similarly be applied to wound-healing experiments with epithelial cells or enhancing specific cellular components such as mitochondria, cell membranes, or nuclei from brightfield microscopic images. Use-case 2 highlights the fine-tuning capabilities of StarDist, applicable to various samples with star-convex shapes, such as T-cells, lipid droplets, and extracellular vesicles, across different imaging modalities like immunohistochemistry and electron microscopy. In addition, other models like Omnipose or CellPose could be fine-tuned for specific experimental needs. Use-case 3 focuses on analyzing large images, a workflow that can be adapted to whole-slide imaging in digital pathology or high-resolution brain section imaging. These examples underscore the adaptability of deepImageJ for diverse biological contexts, enhancing its utility across different research scenarios.

Looking ahead, a key area of focus for future work is the integration of interactive annotation features. This functionality will enable users to fine-tune DL models directly within the deepImageJ environment. By incorporating tools for interactive annotations, researchers will have the flexibility to customize and refine their models with greater precision and ease, tailoring them to specific research needs. This addition is particularly significant for cases where standard pre-trained models may not perfectly align with unique data set characteristics, allowing for more personalized and accurate analysis.

Another significant advancement planned is the implementation of a transparent connection with Python. This development will endow deepImageJ with full training capabilities, effectively transforming it into a comprehensive platform for both model development and application. By bridging deepImageJ with Python, a leading language in the field of data science and machine learning, users will gain access to a vast ecosystem of libraries and tools. This integration will not only facilitate the training of DL models within deepImageJ but also enable seamless interoperability between deepImageJ and a wide range of Python-based data processing and analysis frameworks.

The development of the deepImageJ environment and related initiatives represent a paradigm shift in how biologists and researchers approach bioimage analysis. By lowering the barrier to entry for applying advanced DL techniques, deepImageJ democratizes access to cutting-edge analysis methods. This accessibility is vital for fostering a culture of innovation and exploration in the life sciences, where researchers can leverage these tools to uncover new insights into complex biological phenomena. As bioimage analysis continues to evolve, tools like deepImageJ will play a fundamental role in bridging the gap between advanced computational techniques and practical research applications, driving forward the frontiers of science and medicine.

## 5. Conclusions

In conclusion, this manuscript has presented the evolution of deepImageJ, highlighting key advancements and new features. The integration of the JDLL has played a key role in expanding the capabilities of deepImageJ, making it a versatile and accessible tool for life scientists and bioimage analysts. The case studies showcased the practical applications of deepImageJ across different biological scenarios, demonstrating its effectiveness in tasks ranging from cell segmentation to plant tissue analysis.

The introduction of the JDLL has significantly streamlined the execution of DL models, providing a unified framework for various DL engines and frameworks. The ability to run different engines in a single Fiji/ImageJ instance opens up new possibilities for constructing complex image analysis pipelines. The enhanced compatibility with TensorFlow 1 and 2, PyTorch, and ONNX, coupled with the capability to process larger images, marks a significant step forward in the field of bioimage analysis.

The zero-coded nature of deepImageJ, coupled with the new features introduced in version 3.0, underscores its commitment to democratizing access to DL tools for life scientists. This paper not only serves as a comprehensive documentation of deepImageJ’s journey but also aims to inspire the research community to harness the power of DL in the realm of bioimage analysis.

### 6. Materials

### 6.1. Data sets

#### 6.1.1. Case study 1: Lifeact-RFP cells with SiR-DNA

The data sets employed for training in both tasks are publicly available and have been used extensively for similar research in fluorescence microscopy. These data sets consist of images of live cells expressing Lifeact-RFP (Red Fusion Protein) for visualizing actin filaments and are treated with 0.5 *μ*M SiR-DNA for live cell DNA staining. The continuous imaging of cell culture was performed over 14 h using a spinning disk confocal microscope, capturing images at 10-min intervals. This imaging was done with a Yokogawa CSU-W1 scanning unit on an inverted Zeiss Axio Observer Z1 microscope with a x20 (numerical aperture [NA] 0.8) air, Plan-Apochromat objective (Zeiss).^(^^9^^,^^24^^)^

For the Pix2Pix network training, we used pairs of images from these datasets: membrane-stained (Lifeact-RFP) and nuclei-stained (SiR-DNA) images. The same nuclei-stained images were also employed for training the StarDist model.^(^^24^^)^ To generate the mask images, the authors used Fiji/ImageJ^(^^9^^)^ as detailed in their documentation^1^.

#### 6.1.2. Case study 2: Developing T. Castaneum embryo

In the context of Case Study 2, a specialized data set from the Cell Tracking Challenge was utilized to fine-tune the StarDist network (see Supplementary Material for the link to download). This data set includes high-resolution fluorescence microscopy images capturing the developing *T. castaneum* embryo nuclei. The images were acquired using a Zeiss LightSheet LZ.1 microscope equipped with a Plan-Apochromat x20/1.0 (water) objective lens, achieving a voxel size of 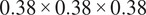 *μ*m. The images were taken at 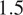 -min intervals to track cellular dynamics over time. For detailed information on sample preparation, RNA injections, and imaging techniques, please refer to Jain *et al.*^(^^25^^)^

It is important to recognize that the image data, acquired using a light-sheet microscope, was fused from multiple viewpoints. Due to this, some views might not align perfectly, leading to the appearance of false or conspicuous nuclei. Furthermore, as not all views cover the entire volume, localized dark patches may be present along the image axis.

The experiment involved two separate embryos. The first embryo (Embryo 01) was used to train the StarDist network in ZeroCostDL4Mic, whereas the second (Embryo 02) served as an independent data set for testing the methodology in Fiji/ImageJ. Crucially, the annotations used for network training are sparse, focusing only on selected regions and cell lineages. This sparsity, particularly in the beetle’s blastoderm at the junction of embryonic and extra-embryonic tissues, was essential for effective network training. The sparse annotations provided a focused and relevant data set for fine-tuning the network, as depicted in Figure 2.

#### 6.1.3. Case study 3: *Arabidopsis* apical stem cells segmentation data set

In this case study, we utilized a publicly available confocal imaging-based data set of plant cells from Willis et al.,^(^^23^^)^ which includes data from six *A. thaliana* plants treated with naphthylphthalamic acid. This treatment inhibits auxin transport, allowing the study of its effects on plant development and physiology.

Confocal z-stacks were acquired every 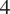 h for 3–3.5 days at a resolution of 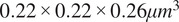 per voxel using a 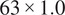 NA water-immersion objective. For each plant, ~ 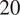 data time points were available. Each time point comprises a stack of around 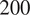 image slices, with each slice measuring 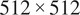 pixels. The data set includes segmentation ground truth; for instance, segmentation of each cell. Specifically, we analyzed an image stack from Plant number 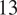 , which displays cell membranes expressing acylYFP in a shoot apical meristem 84 h post-treatment.

## 7. Methods

The three case studies illustrate the versatility of DeepImageJ, demonstrating its applicability to both complex and straightforward workflows. The first two case studies present comprehensive pipelines, including the fine-tuning of one or two networks and the use of a macro script for efficient batch processing of multiple images. These examples highlight the capacity of DeepImageJ to manage intricate tasks involving model training and large-scale data analysis. In contrast, the third case study showcases the seamless integration between the BioImage Model Zoo and DeepImageJ, focusing on a simpler workflow that underscores the ease of using pre-trained models without extensive setup.^2^

### 7.1. Case study 1: Lifeact-RFP cells with SiR-DNA

#### 7.1.1. Pix2Pix for image translation and StarDist for nuclei segmentation

The Pix2Pix model,^(^^17^^)^ integral to Case Study 1 for the task of image-to-image translation from membrane staining (Lifeact-RFP) to nuclei staining (SiR-DNA), underwent rigorous training for 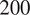 epochs. The training data set consisted of 1748 paired image patches, each with dimensions 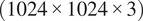 and a patch size of 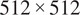 . The training process utilized a batch size of 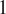 and a vanilla Generative Adversarial Network loss function. Executed within the Pix2Pix ZeroCostDL4Mic notebook (v1.15.1) on Google Colab, the model was trained to adhere to the default parameters of the notebook. No data augmentation was applied during training. Key training parameters encompassed a patch size of 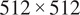 , a batch size of 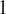 , and an initial learning rate of 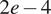 , achieving successful translation from membrane to nuclei staining. The Pix2Pix model is exported using PyTorch 2.0.1.

After the image-to-image translation, the StarDist model, designed for nuclei segmentation, underwent extensive training for 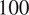 epochs. StarDist consists of a UNet trained to identify the intrinsic features of an object, such as the centroid or oriented distances from the centroid to its boundary, which enable its reconstruction as a star-convex polygon. The 2D variant of StarDist^(^^11^^)^ was trained and evaluated using its implementation within the StarDist 2D ZeroCostDL4Mic notebook (v 1.19). The training data set consisted of 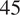 paired image patches, each with dimensions 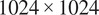 and a patch size of 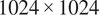 . The training process used a batch size of 2. The model was fine-tuned from a pre-trained model, applying no data augmentation during training. Executed within the Google Colab environment, the training parameters included a patch size of 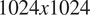 , a batch size of 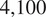 epochs, and an initial learning rate of 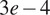 . The resulting StarDist model is exported with Tensorflow 2.14.

#### 7.1.2. Post-processing with StarDist and TrackMate

In Case Study 1, the reconstruction of 2D star-convex polygons is facilitated by the StarDist plugin for Fiji/ImageJ, which supports macro recording in ImageJ. Therefore, an ImageJ/Fiji macro^3^ is utilized to execute the complete pipeline, encompassing the running of Pix2Pix, StarDist, and the subsequent StarDist PostProcessing. Following the generation of masks by this pipeline, the trajectories of cells across five available time points are analyzed using TrackMate.^(^^19^^)^ The results, including the cell trajectories, are illustrated in Figure 1.

### 7.2. Case study 2: Developing T. Castaneum embryo

#### 7.2.1. Preprocessing

In Case Study 2, preprocessing steps are essential before inputting images into the StarDist network. It is important to note that for fine-tuning the ZeroCostDL4Mic model, we utilize sparse annotations of the beetle’s blastoderm, as described in Section 6.1.2. Consequently, only selected slides from the data set are employed. However, during the inference process in deepImageJ, the entire volume corresponding to each time point is processed.

A two-stage preprocessing strategy is implemented to address the image noise and reduce the computational load. The first step involves applying a median filter across all images to reduce noise effectively. Following this, a downsampling operation is conducted. This operation reduces the resolution by half along the 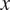 and 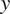 axes for the slices used in fine-tuning the ZeroCostDL4Mic model and along all three axes (*x*, *y*, and *z*) when processing the entire volume for inference with deepImageJ.

#### 7.2.2. StarDist: Nuclei segmentation in 2D

The segmentation network employed in Case Study 2 is based on StarDist,^(^^11^^)^ a deep-learning method designed to precisely segment cell nuclei from bioimages. This method uses a shape representation founded on star-convex polygons to predict both the presence and shape of nuclei within an image. The 2D variant of StarDist relies on an adapted UNet architecture, allowing for efficient segmentation of 2D data sets. Implemented within the ZeroCostDL4Mic framework, the StarDist 2D model was specifically tailored for nuclei segmentation within the context of the “Developing *T. castaneum* embryo” data set, as described in Section 6.1.2. The data set structure was adjusted accordingly, to facilitate compatibility with the notebook’s data reading mechanism. The code detailing the data structuring process is available on the deepImageJ GitHub for reproducibility purposes. Data set augmentation was performed by a factor of four via random rotations, flips, and intensity changes.

The training regimen involved 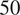 epochs on 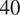 paired image patches of size 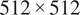 cropped from the original images ( 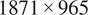 pixels). A batch size of 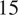 was utilized, employing a Mean Absolute Error/L1 loss function. The model was retrained from a preexisting pre-trained model (2D Versatile fluo from StarDist Fiji), with key training parameters including a learning rate of 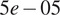 , 10% validation data, 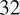 rays (n_rays), and a grid parameter of 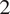 . Despite challenges associated with ground truth variability, particularly in cases where only one nucleus is marked in the mask, the model demonstrated good performance. This effectiveness was observed in the segmentation accuracy, where the predicted results were consistently aligned with the available ground truth, despite its inherent variability.

#### 7.2.3. Post-process with StarDist

In Case Study 2, the post-processing of StarDist is streamlined through an ImageJ macro, which processes each slice of the 3D embryo volume independently. The macro, designed to handle 488 slices per timepoint, applies the StarDist model slice by slice. For each slice, the StarDist model is applied, followed by a series of post-processing operations. These operations, essential for accurate object detection and minimizing overlap, include applying specific thresholds for probability and non-maximum suppression. The macro utilizes the StarDist plugin to ensure precise segmentation results for each 2D slice.

This macro effectively transforms the multichannel output of StarDist into a single, comprehensive mask. In the final phase, the Connected Components algorithm is applied across the entire 3D volume. This process results in a detailed visualization of the entire volume, with each segmented cell clearly delineated, as illustrated in Figure 2.

### 7.3. Case study 3: *Arabidopsis* apical stem cells segmentation

#### 7.3.1. 3D UNet *Arabidopsis* apical stem cells segmentation

In Case Study 3, a 3D UNet^(^^22^^)^ was employed for cell boundary segmentation. This pre-trained model is accessed from the bioimage.io website for inference, and it is also available through Zenodo^4^.

The authors of the network^(^^22^^)^ employed a training strategy where the 3D UNet was trained on ground truth cell contours obtained by applying a Gaussian blur to a two-voxel-thick boundary between labeled regions. The training regimen featured a combination of binary cross-entropy and Dice loss, with notable architectural modifications, including replacing batch normalization with group normalization and utilizing the same convolutions instead of valid convolutions. During training, augmentation techniques such as flips, rotations, elastic deformations, and noise augmentations were employed, to enhance the model’s generalization capabilities.

The trained model is available on bioimage.io under the emotional-cricket, allowing accessibility for the wider research community. In this case study, the network is exclusively employed for inference on the specified dataset.

#### 7.3.2. Postprocess

In the post-processing phase of Case Study 3, the pipeline includes two distinct steps. Initially, a Gamma correction function, set at 0.80, is applied to enhance membrane visibility and reduce any blurriness. Subsequently, the Morphological Segmentation tool from MorpholibJ^(^^20^^)^ is utilized for both segmentation and visualization. This tool is employed with a tolerance setting of 10, enabling the effective depiction of catchment and overlay basins on the segmented image. This precise application of Morphological Segmentation ensures clear and distinct visualization of each cell.

## Supporting information

Fuster-Barceló et al. supplementary material Fuster-Barceló et al. supplementary material

## Data Availability

To ensure the reproducibility of our study, we have made all data, code, and models accessible in various formats. The data sets are accessible from the dedicated repositories specified in the Data sets section. For models fine-tuned during the training process, they are accessible in the BioImage Model Zoo and Zenodo, and the corresponding notebooks used for fine-tuning are available on ZeroCostDL4Mic. Additionally, all codes used for data set construction, ImageJ Macros, and other relevant tools are accessible in a GitHub repository associated with deepImageJ. All links and details are also specified in the Supplementary Material as well as in the GitHub repository dedicated to it at https://github.com/deepimagej/case-studies.

## Footnotes

1
https://github.com/HenriquesLab/ZeroCostDL4Mic/wiki/Stardist#generating-masks-for-stardist-2d

2 For reproducibility purposes, a step-by-step guide for each case study is included in the deepImageJ documentation.

3 The macro utilized for this process can be accessed in our case studies documentation at https://github.com/deepimagej/case-studies

4 3D Unet *Arabidopsis* apical stem cells model in Zenodo https://zenodo.org/records/7768142

## Supplementary material

The supplementary material for this article can be found at http://doi.org/10.1017/S2633903X24000114.
