# Supplementary material for: Bridging the gap: Integrating cutting-edge techniques into biological imaging with deepImageJ
Source: Biol Imaging. 2024 Nov 22;4:e14. doi: 10.1017/S2633903X24000114 (PMC11704127; doi:10.1017/S2633903X24000114)
Supplement: Fuster-Barceló et al. supplementary material [file S2633903X24000114sup001.docx]

## Supplementary Material: Data, Notebooks, Code, and Models Availability

The following table provides details on the datasets, notebooks, code, and models used in each case study. This information is essential for ensuring reproducibility and is included in the supplementary material.

| **Case Study** | **Resource Type** | **Description and Links** |
| --- | --- | --- |
| Case Study 1 | Files | *prepare_dataset.py*, *StarDist Postprocess macro CS1.ijm* |
|  | Notebooks | Pix2Pix Notebook, StarDist 2D Notebook |
|  | Dataset | Lifeact-RFP, sir-DNA DAPI |
|  | Models | Pix2Pix Model, StarDist Model |
| Case Study 2 | Files | *Generated_GT.py*, *Mount_stardist_dataset.py*, *StarDist_postprocess_macro_cs2.ijm* |
|  | Notebooks | StarDist 2D Notebook |
|  | Dataset | Developing Tribolium Castaneum Embryo |
|  | Model | StarDist Model |
| Case Study 3 | Dataset | Arabidopsis Apical Stem Cells |
|  | Model | 3D Unet Arabidopsis Model |

***Table 1.*** *Availability of data, notebooks, code, and models for each case study.*

Additionally, all codes related to these case studies can be found in the GitHub repository ImageJ Case Studies. Also, a detailed explanation in a step by step guide to reproduce all steps of the case studies can be found in the https://github.com/deepimagej/deepimagej-plugin/wiki/Example-Usage-and-Tutorials.
